# Effect of the Type VI Secretion System Secreted Protein Hcp on the Virulence of *Aeromonas salmonicida*

**DOI:** 10.3390/microorganisms10122307

**Published:** 2022-11-22

**Authors:** Hongyan Cai, Jiaying Yu, Ying Qiao, Ying Ma, Jiang Zheng, Mao Lin, Qingpi Yan, Lixing Huang

**Affiliations:** 1 Key Laboratory of Healthy Mariculture for the East China Sea, Fisheries College, Ministry of Agriculture, Jimei University, Xiamen 361021, China; 2 Fourth Institute of Oceanography, Ministry of Natural Resources, No. 26, New Century Avenue, Beihai 536000, China

**Keywords:** *Aeromonas salmonicida*, type-VI secretion system, *hcp*, virulence

## Abstract

*Aeromonas salmonicida*, a psychrophilic bacterial pathogen, is widely distributed in marine freshwater, causing serious economic losses to major salmon farming areas in the world. At present, it is still one of the most important pathogens threatening salmon farming. Hcp (haemolysin-coregulated protein) is an effector protein in the type-VI secretion system (T6SS), which is secreted by T6SS and functions as its structural component. The results of our previous genomic sequencing showed that *hcp* existed in the mesophilic *A. salmonicida* SRW-OG1 isolated from naturally infected *Epinephelus coioides*. To further explore the role of Hcp in *A. salmonicida* SRW-OG1, we constructed an *hcp*-RNAi strain and verified its effect on the virulence of *A. salmonicida*. The results showed that compared with the wild strain, the *hcp*-RNAi strain suffered from different degrees of decreased adhesion, growth, biofilm formation, extracellular product secretion, and virulence. It was suggested that *hcp* may be an important virulence gene of *A. salmonicida* SRW-OG1.

## 1. Introduction

*Aeromonas salmonicida* is a facultative anaerobic Gram-negative bacteria. It has a wide host range and is distributed all over the world [1]. In 1894, *A. salmonicidal* was first isolated from Bavarian diseased brown trout (*Salmo trutta*) by Emmerich et al. [2]. Subsequently, more and more scientists reported cases of aquatic diseases caused by A. salmonicida. For example, *A. salmonicida* can infect fish such as rainbow trout (*Oncorhynchus mykiss*), Atalntic salmon (*Salmo salar*), black fin reef shark (*Carcharhinus melanopterus*), flounder (*Paralichthys olivaceus*), and turbot (*Scophthalmus maximus*). About one week after infection, the case fatality rate reaches 100% [3,4,5,6]. Moreover, *A. salmonicida* can also cause mixed infections in fish together with other species of *Aeromonas* [7,8]. The high mortality rate of *A. salmonicida*-infected fish has caused huge economic losses to the aquaculture industry. Therefore, in order to reduce the harm caused by *A. salmonicida* to aquaculture, it is urgent to study the pathogenic mechanism of *A. salmonicida*.

The pathogenic effect of *A. salmonicida* is closely related to the various virulence factors it carries. Existing studies have shown that the virulence factors of *A. salmonicidal* mainly arise from the type-III secretion system, extracellular polymeric substances (EPSs), siderophores, quorum sensing, and S-layer proteins [9,10,11,12]. At the same time, the secretion of many important virulence factors of bacteria is related to the secretion system. The secretion system can help bacteria to transfer proteins into the external environment or host cells through the bacterial membrane. For example, the-type III secretion system can play a pathogenic role by secreting proteins or directly injecting the virulence proteins into host cells [13]. In addition, studies have shown that the type-VI secretion system (T6SS) also plays an important role in the pathogenic mechanism of Gram-negative bacteria [14]. T6SS is a complex transmembrane nanomachine composed of multiple proteins, and most bacteria contain multiple sets of T6SS [15]. T6SS comprises 13 core elements, which can be divided into three main parts: a transmembrane complex composed of TssJ/L/M; a wedge complex composed of TssE/F/G/K; and Hcp (haemolysin-coregulated protein), a TssB/C phage tail structure with VgrG [16]. The T6SS not only participates in population competition between bacteria, but also has the function of killing xenogeneic bacteria and is related to bacterial virulence [17], which plays an important role in the regulation of the pathogenicity of bacteria.

Hcp is an important pipeline structural protein in T6SS. It belongs to the same super-protein family as VgrG and is the structural basis for the function of T6SS [18]. Hcp has a hollow hexameric tubular structure, consisting of six monomeric proteins connected in a head-to-head or head-to-tail manner to form hollow ring structures, which are then stacked layer by layer through disulfide bonds to form an inner diameter of 35–40 Ao. Circular hollow hexamers with an outer diameter of 80–90 Ao can transport some small folded (<20 KDa) or partially unfolded proteins through the intermediate pore to the environment or target cells [19]. Hcp is not only the structural protein of T6SS, but it also plays an important role as an effector protein in bacterial pathogenic mechanisms. For example, Hcp effector proteins of various bacteria, such as *A. hydrophila*, *Vibrio cholerae*, and *Salmonella*, can affect various processes, such as bacterial adhesion and invasion, survival in phagocytes, host immune response, and pathogenicity [20,21].

At present, many studies have reported the pathogenic role of Hcp in T6SS in *A. hydrophila* [21], *V. cholerae* [22], *Escherichia coli* [23], and *Acinetobacter eel* [24], but little is known about its functions in *A. salmonicida*. Through our previous genomic sequencing, Hcp was found to exist in the mesophilic *A. salmonicida* SRW-OG1 isolated from naturally infected orange-spotted grouper (*Epinephelus coioides*) [25]. According to our RNA-seq results, we found that the expression level of *hcp* in *A. salmonicidal* was significantly affected by water temperature. However, there are no previous reports on the involvement of Hcp in regulating virulence in *A. salmonicidal*, especially the effect of its response to temperature on the pathogenic process. In view of the importance of Hcp in T6SS and the bacterial infection process, the present study aimed to investigate the relationship between Hcp and *A. salmonicidal* virulence. It is of great significance to study the virulence regulation mechanism of *A. salmonicidal* based on *hcp* for the prevention and treatment of *A. salmonicidal* infection.

## 2. Materials and Methods

### 2.1. Strains and Culture Conditions

*A. salmonicida* SRW-OG1 strain was isolated from the lesions of diseased *E. coioides* in Zhangzhou City, Fujian, China in 2018 [26]. It was stored in a freezer at −80 and inoculated in Luria Bertani (LB) broth for extended culture at 18 °C and 220 rpm. *E. coli* DH5α was obtained from TransGen Biotech (Beijing, China), inoculated in LB broth, and cultured at 37 °C and 220 rpm.

### 2.2. Sequence Alignment Analysis, Phylogenetic Tree Construction, and Protein Structure Prediction

In order to study the similarity of Hcp among species, the amino acid sequence of the Hcp was analyzed. Eleven Hcp sequences of *Aeromonas*, *Vibrio*, and *Pseudomonas* were selected, and a phylogenetic tree was constructed by the neighbor-joining method (NJ method) [27]. The amino acid sequence of the Hcp of the *A. salmonicida* SRW-OG1 strain was compared with that of *A. salmonicida* (strain: O23A), *Plesiomonas shigelloides* (strain: 7A), *V. cholerae* (strain: RFB16), *Vibrio parahaemolyticus* (RIMD 2210633), *Proteus mirabilis* (HI4320), *Pseudomonas lactis* (strain: SS101), *Vibrio harveyi* (strain: ATCC 33843 (392 [MAV])), *Vibrio anguillarum* (strain: PF4-E2-R4), *Vibrio alginolyticus* (strain: FDAARGOS-97), *Pseudomonas fluorescens* (strain: ATCC 13525), and *Vibrio mimicus* (MB451) by ClustalW and DNAMAN Version 9.0 to map the sequence alignment results; MEGA7.0 software was used to construct the phylogenetic tree. The protein structure of the Hcp of *A. salmonicida* was predicted and analyzed by I-TASSER [28]; the process of I-TASSER to achieve structure prediction and functional labeling comprises four main steps: (1) the identification of templates by LOMETS [29]; (2) fragment structure assembly using a copy–swap Monte Carlo algorithm [30]; (3) atomic-level structure refinement using REMO [31] and FG-MD [32] (the model after FG-MD refinement is the final model for tertiary structure prediction output by I-TASSER; the quality of the final model is estimated by the C-score value); (4) the derivation of the structure-based protein function using COFACTOR [33] (a three-dimensional all-atom model obtained by FG-MD is used to predict protein functions, including EC (enzyme commission) number [34] and ligand-linking domain (binding site)).

### 2.3. Quantitative Real-Time Polymerase Chain Reaction (qRT-PCR)

First, total RNA was extracted from bacterial cells with TRIzol reagent (Invitrogen, Carlsbad, CA, USA) according to the manufacturer’s instructions. The TransScript One-Step gDNA Removal and cDNA Synthesis SuperMix kit (TransGen Biotech, Guangzhou, China) was used to carry out reverse transcription from 20 μg total RNA, as instructed by the manufacturer [35]. Second, qRT-PCR was performed using a QuantStudio 6 Flex real-time PCR system (Life Technologies, Carlsbad, CA, USA) with the Power Green qPCR Mix (Guangzhou Dongsheng Bio, Guangzhou, China). The total reaction volume was 10 μL, which included 0.25 μL forward primer (10 μM), 0.25 μL reverse primer (10 μM), 0.5 μL template, and 9.0 μL 2 × TransStart Top Green qPCR SuperMix. The thermal cycler conditions were 95 °C for 2 min, followed by 40 cycles of 95 °C for 20 s, 58 °C for 20 s, and 72 °C for 20 s. All qRT-PCR experiments were performed in triplicate using independent samples. The 2^−ΔΔCt^ method was applied for analysis [36,37]. *16S* rRNA was used to normalize the gene expression levels. Primers were synthesized by Xiamen Borui Biotechnology Co., Ltd. (Xiamen, China). All primers are listed in Table S1.

### 2.4. Construction of hcp Silent Strains

RNAi strains were constructed based on a previously described method [38,39] with minor modifications. In brief, five short hairpin RNA (shRNA) sequences for *hcp* were designed using Block-IT™ RNAi Designer, and the shRNA sequences were synthesized by Shanghai General Biotechnology Co., Ltd. (Shanghai, China). pCM130/tac vectors were linearized using restriction endonuclease *NsiI* HF and *BsrGI* HF (New England Biological Laboratory, Franklin Lakes, NJ, USA), as recommended by the manufacturer. The shRNA segments were ligated into the plasmid pCM130/tac using T4 DNA ligase (New England Biological Laboratory, Franklin Lakes, NJ, USA). The recombinant pCM130/tac vector was transformed into *E. coli* DH5α competent cells by the heat shock method. An EasyPure plasmid MiniPrep Kit (TransGen Biotech, Beijing, China) was used to extract plasmid DNA from *E. coli* DH5α cells, which was then transferred into *A. salmonicida* via the electrical transfer method. Finally, as mentioned above, quantitative real-time polymerase chain reaction (qRT-PCR) was used to detect the *hcp* expression level of each RNAi strain, and that with the best silencing efficiency was used for further studies. The primer sequences are listed in Supplementary Materials Table S1.

### 2.5. Growth Curve Measurement

According to the description of Zuo et al. [40], the *hcp*-RNAi and wild-type strains of *A. salmonicida* were inoculated into fresh LB broth containing tetracycline (10 μg/mL) and LB broth without antibiotics, respectively, and cultured overnight in a 220 rpm biochemical incubator at 18 °C. The bacterial solution was adjusted to 1.0 × 10^8^ CFU/mL; then, 10 μL bacterial culture solution was mixed with 190 μL LB broth in each well of the 96-well plate. The concentration of the bacterial solution was measured hourly for 24 h, and the OD_600_ nm absorption was recorded. Growth curves were drawn based on absorbance at OD_600_ nm, with 6 replicates per group.

### 2.6. Biofilm Formation Measurement

Following Hu et al. [41], *A. salmonicida* was transferred to LB broth, grown at 18 °C overnight, and then adjusted to 1.0 × 10^8^ CFU/mL. One hundred microliters of bacterial solution was added to each well on a 96-well plate and cultured at 18 °C for 24 h. The cultures were then washed with sterile PBS twice, dried aseptically, incubated with 200 μL crystal violet (0.1%) for 15 min, washed with sterile PBS again, and finally air-dried. The stained biofilm was dissolved with 200 μL 33% acetic acid and quantified by OD_590_ nm. Six independent biological replicates were performed for each data point.

### 2.7. Preparation of Mucus

Healthy *E. coioides* were obtained from the City of Xiamen (Fujian, China). The method of mucus preparation followed Mao et al. [42], with a slight modification. Briefly, the surface of the fish was rinsed with sterile PBS, and the mucus on the surface of the fish was gently scraped off with a glass slide and mixed with PBS. This mixture was then centrifuged at 2000 rpm for 30 min (twice) at 4 °C, and the supernatant was sequentially filtered through 0.45 μm and 0.22 μm pore-size filters. Finally, the mucus concentration was adjusted to 1 mg/mL in PBS using Bradford’s method.

### 2.8. Adhesion Test

The adhesion ability was detected according to the method of Huang et al. [43], though some adjustments were made. Twenty-microliter sterile mucus drops were placed in the center of a 22 × 22 mm^2^ slide and evenly spread. After the slime on the slide was dried naturally, it was fixed with 4% methanol for 30 min. Two hundred microliters of bacterial resuspension (1.5 × 10^8^ CFU/mL) was applied to the slime area. The slides were incubated at 18 °C for 2 h, then rinsed with sterile PBS 5 times, air-dried, and fixed with 4% methanol again for 30 min. Two hundred microliters of crystal violet solution (0.1%) was then used to stain the slides for 3 min. The crystal violet solution was washed off with PBS, and then the stained bacterial cells were observed under a microscope (×1000). The average number of bacteria adhering to the glass surface within the visual field was counted. For each analysis, 20 fields were calculated.

### 2.9. Hemolysis Test

The hemolysis activity was detected using the method described by He et al. [44], with slight modifications. First, the wild-type strain of *A. salmonicida* and the *hcp*-RNAi strain were cultured at 18 °C and 200 rpm, then adjusted to 1.5 × 10^8^ CFU/mL after culturing to a stationary phase. Next, 1.2 mL of the bacterial solution was added to a 1.5 mL centrifuge tube and centrifuged at 12,000 rpm for 1 min at room temperature; then, 245 μL of the supernatant was transferred to a new centrifuge tube. One hundred microliters of sheep blood purchased from Pingrui Biotechnology Co., Ltd. (Beijing, China) was washed three times with PBS. Finally, 5 μL of washed sheep blood was mixed with 245 μL of culture supernatant and incubated at 37 °C with shaking (150 rpm) for 1 h. After incubation, samples were centrifuged, and the amount of released hemoglobin was determined according to the OD_540_. The percentage of total hemolysis was calculated by comparing the OD_540_ for the positive (ddH_2_O) and negative control (PBS) samples. Six independent biological replicates were performed for each data point.

### 2.10. Artificial Infection and Sample Collection

All animal experiments were carried out following the ‘Guide for the Care and Use of Laboratory Animals’ published by the Animal Ethics Committee of Jimei University (Acceptance No. JMULAC201159).

Healthy *E. coioides* (50.0 ± 2.0 g) were randomly divided into groups (*n* = 40/group, 3 repeated experiments). The *A. salmonicida* wild-type and *hcp*-RNAi strains were cultured at 18 °C, 200 rpm. The bacterial concentration was adjusted to 1.0 × 10^3^ CFU/mL [45]. Two hundred microliters of bacterial solution was injected at the base of the pectoral fin of the fish, and the negative control was injected with the same amount of PBS and observed daily.

To determine the expression level of *hcp* during infection, 3 *E. coioides* spleens each were extracted 1d, 2d, 3d, and 4d after the injection of the wild-type strain. For bacterial colonization assessment, three spleens were obtained at 1, 6, 12, 24, 48, 72, and 96 hpi from *E. coioides* injected with wild-type and *hcp*-RNAi strains, respectively [46]. The RNA of the spleen tissue was extracted and reverse-transcribed; finally, the gene expression was detected by qRT-PCR.

### 2.11. Preparation of Extracellular Products

Extracellular polymeric substances (EPSs) were prepared by the plate cellophane method with minor modifications [47]. First, the bacteria were transferred into LB broth and cultured in a biochemical incubator at 18 °C and 120 r/min for 24 h. Sterile cellophane was spread on the TSA plate, and 200 μL of the bacterial suspension was evenly spread on the TSA plate and cultured at 18 °C for 48 h. After incubation, the cultures were washed with 4 mL sterile PBS, collected, and centrifuged at 4 °C and 12,000 r/min for 20 min. The supernatant was extracted and centrifuged again for 10 min, frozen to a solid state in a −80 °C freezer for 48 h, and then transferred to a vacuum freeze-dryer to produce a powder. Finally, the milky-white powdered sample was dissolved with sterile PBS, filtered using a 0.22 μm filter membrane, and sterilized, producing the crude extracellular product. The concentration of protein samples in the extracellular products was determined using Bradford’s method [48].

### 2.12. SDS-PAGE Electrophoresis

In order to explore the effect of the *hcp* gene on the protein composition of *A. salmonicida* extracellular products, we measured the protein composition of the *A. salmonicida* extracellular products before and after *hcp* gene silencing by SDS-PAGE electrophoresis. Referring to the method of Sebastian et al. [49], 25 μL of the sample (extracellular product: 5 × SDS-PAGE loading buffer = 4:1) was used for the electrophoresis. After electrophoresis, the gel was stained with 1 × Coomassie brilliant blue solution for 15 min; washed with 40 mL of decolorizing solution (glacial acetic acid: ethanol: double-distilled water = 1:0.5:8.5); heated for 25 s; decolorized for 3 h; and imaged using a JS-680D Automatic Gel Imaging System Analyzer (Pei Qing, China) after decolorization.

### 2.13. Effect of hcp Gene on the Enzyme Activity of A. salmonicida Extracellular Products

The determination method was based on Yang et al. [50], with slight modifications. Agar plates were prepared with distilled water containing sterile casein (0.4%), skim milk powder (0.4%), Tween 80 (1.0%), and sterile egg yolk liquid (2.5%). Twenty microliters of the prepared extracellular product samples was added to the agar plate after drilling, and the same amount of normal saline (0.85%) was used as negative control. After incubation at 28 °C for 36 h, 10 μL of trichloroacetic acid solution (10%, *w*/*w*) was added to casein and skim milk powder agar plates. The detection of transparent circles (casein and skim milk powder agar plates) or opaque circles (Tween 80 and egg yolk agar plates) around the holes was identified as a positive reaction.

### 2.14. Preparation and Determination of Extracellular Polysaccharide and Protein in EPSs

The ultrasonic method was used to extract EPSs from bacterial biofilms, with a slight modification [51]. Briefly, bacteria were inoculated in LB liquid medium and cultured for 48 h (18 °C, 120 r/min). At the end of the culture period, the optical absorbance of OD_595_ was measured using a SYNERGYH1 multifunctional microplate reader (BioTek, Winooski, VT, USA). The medium was removed and washed 3 times with sterile PBS to remove bacteria that had not yet formed a biofilm. One microliter of 0.01 mol/L KCl solution was added to resuspend the bacteria for 5 min. Then, each sample hole was sonicated for 5 s with an interval of 5 s. This cycle was repeated 5 times. After sonication, the bacterial solution was transferred to a 1.5 mL sterile centrifuge tube and centrifuged at 4000 r/min and 4 °C for 20 min. Then, a 0.22 μm filter membrane was used to filter the sample into a new centrifugal tube for later use.

The relative content of extracellular proteins in the EPSs was determined with Lowry reagent. First, we added 200 μL of Lowry reagent to a 1.5 mL sterile centrifuge tube, then added 40 μL of the filtered sample, mixed gently, and incubated the mixture at room temperature for 10 min. Twenty microliters of Folin phenol reagent was added, and after incubation at room temperature for 30 min, the value of the sample at OD_750_ nm was measured using a SYNERGYH1 multifunctional microplate reader (BioTek, USA). Finally, the relative content of extracellular proteins in the biofilms was calculated by the OD_750_/OD_595_ method.

The relative content of exopolysaccharides in the EPSs was determined by the phenol-sulfuric acid method. First, we placed 100 μL of the filtrate sample into a 1.5 mL sterile centrifuge tube, then added 200 μL of 98% concentrated sulfuric acid with a pipette, mixed gently, and incubated the mixture at room temperature for 30 min. Next, we added 25 μL of 6% phenol solution, mixed gently, heated the metal bath at 90 °C for 5 min, and extracted the mixture. The value of the sample at OD_490_ nm was measured using a SYNERGYH1 multifunctional microplate reader (BioTek, USA), and the relative content of exopolysaccharide in the biofilm was calculated by the OD_490_/OD_595_ method.

### 2.15. Statistical Analysis

Data are presented as mean ± standard deviation (SD) and were assessed using SPSS 18.0. Differences were determined by one-way ANOVA followed by Dunnett’s multiple comparisons test. *p* < 0.05 indicated statistical significance.

## 3. Results

### 3.1. Phylogenetic Tree and Protein Structure Analysis of Hcp from A. salmonicida

**Figure 1:**
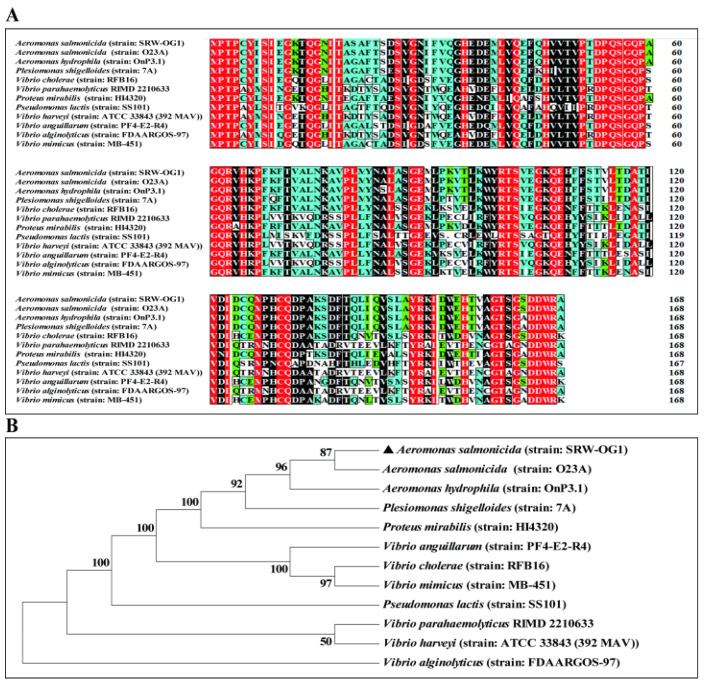
Phylogenetic tree and sequence alignment of the amino acid sequence of the *hcp* gene. (**A**) Amino acid sequence alignment. (**B**) Phylogenetic tree. Note: numbers at each branch indicate the percentage bootstrap values on 1000 replicates.

The analysis of the amino acid sequences showed that the amino acid sequence of the Hcp from *A. salmonicida* (strain: SRW-OG1) was 97.67% identical to that of *A. hydrophila* (strain: OnP3.1). The amino acid sequence identity with *P. shigelloides* (strain: 7A) and *V. cholerae* (strain: RFB16) Hcp was also higher than 70% (Figure 1A). The neighbor-joining method in Mega7.0 software was used for the phylogenetic analysis of the bacterial Hcp amino acid sequence, and the default Poisson model was adopted. The phylogenetic tree results showed that *A. salmonicida* (strain: SRW-OG1), *A. salmonicida* (strain: O23A), and *A. hydrophila* (strain: OnP3.1) belonged to the same branch and were the closest relatives (Figure 1B).

**Figure 2:**
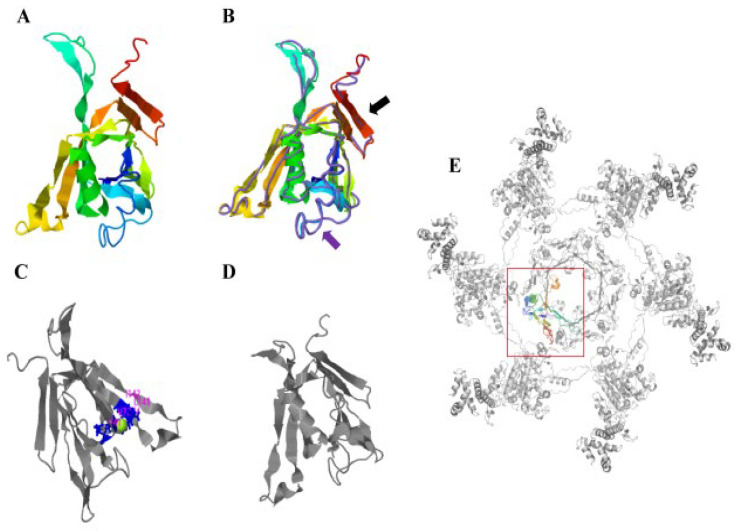
Protein structure prediction of *hcp* gene of *A. salmonicida*. (**A**) Protein structure diagram of Hcp predicted by I-TASSER. (**B**) The crystal structure comparison between the Hcp domain (indicated by the black arrow) of *A. salmonicida* and the original Hcp (PDB ID: 5mxn1) template (indicated by the purple arrow). (**C**) The prediction of ligand-binding sites (ligand-binding residues were V41, T71, V72, L141, and I142). (**D**) Active site map. (**E**) The location distribution of PDB-predicted protein model of Hcp in type-VI secretion system (top view of one ring of Hcp with associated VipA/VipB; the attachment helix of VipB comes into contact with the Hcp ring at the edge of the hexamer).

I-TASSER was used to predict the protein structure by implementing the SPICKER program to cluster all decoys based on paired structural similarity, resulting in the identification of the five largest structural clusters. Of the five largest structural clusters, we chose the cluster with the highest confidence, i.e., a C-score = 1.11 and estimated RMSD = 2.9 ± 2.1 Å. (Figure 2A). The protein structure model of the structural cluster with the highest C-Score value was matched with all the structures in the PDB, and the top 10 proteins with the most similar structures were obtained. According to the highest TM score, we selected the protein with the closest structural similarity to the PDB prediction protein model (PDB ID: 5mxn1), which was the Hcp of *V. cholerae* (Figure 2B). Ligand binding site analysis showed the highest confidence prediction result (C-score = 0.11, Figure 2C). Based on the I-TASSER structure prediction, we used COFACTOR and COACH to predict the structure of the *A. salmonicida* Hcp and the preservation state of the active site of the Hcp. The results showed that the prediction results with the highest confidence were obtained by the enzyme committee (EC) number and enzyme active site analysis of the Hcp (C-score = 0.256; PDB ID = 1nrgA; Figure 2D). In the PDB, the Hcp structure model of *A. salmonicida* (strain: SRW-OG1) was closest to the known protein structure model of *V. cholerae* Hcp. When one protein is structurally highly similar to another, the two proteins tend to have similar functions in relation to their target. Therefore, we predicted that the Hcp of *A. salmonicida* (SWR-OG1) may be functionally similar to that of *V. cholerae*.

### 3.2. Expression Level of Hcp Gene during Infection of E. coioides

**Figure 3:**
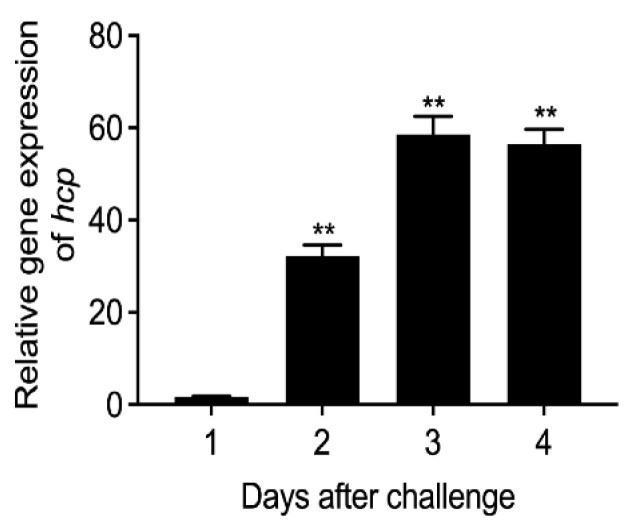
Changes in *hcp* gene expression level during the infection of *E. coioides* with *A. salmonicida*. Data are shown as mean ± SD from three independent biological replicates. ** *p* < 0.01.

In order to detect the expression level of the *hcp* gene of *A. salmonicida* during *E. coioides* infection, we used qRT-PCR technology to detect the expression level after artificially infecting the *E. coioides*. The results showed that the expression level of *hcp* in the spleens of *E. coioides* was low at 24 h after infection (Figure 3). When the infection time was extended to 48 h, the expression of *hcp* increased significantly. Furthermore, when the infection time was extended to 72 h and 96 h, the expression of the *hcp* gene reached its maximum level. The results showed that when fish were infected with *A. salmonicida*, as the infection time increased, the expression of *hcp* also increased significantly. Therefore, it seemed that *hcp* might be involved in the pathogenic process of *A. salmonicida*. However, many genes could change during the infection. The increase in *hcp* expression in the fish host could also be a result of the infection, rather than a cause. Further study is still necessary to validate this finding.

### 3.3. Effects of Hcp Gene Silencing on the Virulence of A. salmonicida

**Figure 4:**
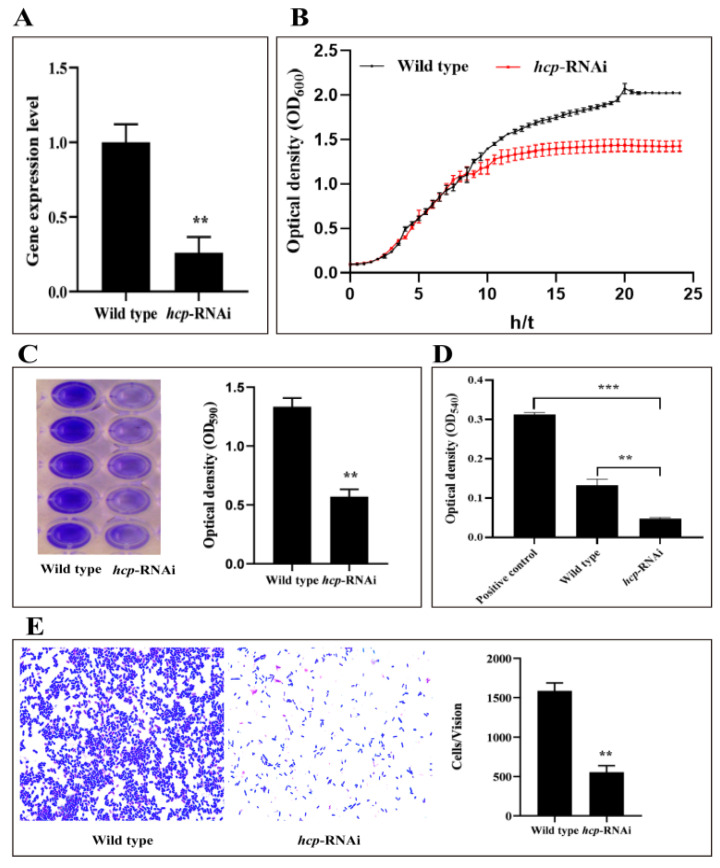
Effects of *hcp* gene silencing on the virulence phenotype of *A. salmonicida*. (**A**) Expression levels of *hcp* in wild-type and *hcp*-RNAi strains identified by qRT-PCR. Data are shown as mean ± SD from three independent biological replicates. (**B**) Effects of *hcp* gene silencing on the growth of *A. salmonicida*. Data are shown as mean ± SD from six independent biological replicates. (**C**) Determination of biofilm-forming ability. (**D**) Determination of hemolysis. (**E**) Determination of adhesion ability. Data are presented as mean ± SD. Three independent biological replicates were performed per group. ** *p* < 0.01, *** *p* < 0.001.

In order to validate the effect of *hcp* on the virulence of *A. salmonicida*, a stable silent strain of *hcp* was constructed, and its silencing effect was verified, as shown in Figure 4A. The results showed that compared with the wild-type strain, the expression level of the *hcp* gene in *hcp*-RNAi decreased by 78.8%, indicating that the stable silent strain of the *hcp* gene was successfully constructed and could be used for subsequent studies.

In order to explore the effect of *hcp* on the growth capacity of *A. salmonicida*, the growth of the wild-type strain and *hcp*-RNAi strain were compared (Figure 4B). The growth rate of the *hcp*-RNAi strain before entering the plateau phase was basically the same as that of the wild-type strain. However, the *hcp*-RNAi strain entered the stationary phase earlier than the wild-type strain (the difference began to appear after the growth of the two strains entered the stationary phase (11 h), and it became significant after 15 h). The results of this experiment showed that *hcp* might play a role in the late growth stage of *A. salmonicida*, but further study is required to illustrate the specific mechanisms involved.

We examined the OD_590_ to determine the biofilm-forming ability of the wild-type and *hcp*-RNAi strains. As shown in Figure 4C, the biofilm-forming ability of the *hcp*-RNAi strain was significantly reduced compared with the wild-type strain. This indicated that the regulation of *A. salmonicida* virulence by the *hcp* gene might be achieved by the modification of its biofilm formation.

Figure 4D displays the determination of the hemolytic ability of the *A. salmonicida* wild-type strain and *hcp*-RNAi strain. The results showed that the hemolytic ability of the *hcp*-RNAi strain was significantly decreased compared with the wild-type strain. This indicated that the *hcp* gene might be involved in the regulation of the hemolysis-related mechanisms of *A. salmonicida*.

As shown in Figure 4E, compared with the wild-type strain, the adhesion ability of the *hcp*-RNAi strain was significantly weakened. This indicated that the *hcp* gene plays a key role in regulating the adhesion ability of *A. salmonicida*.

**Figure 5:**
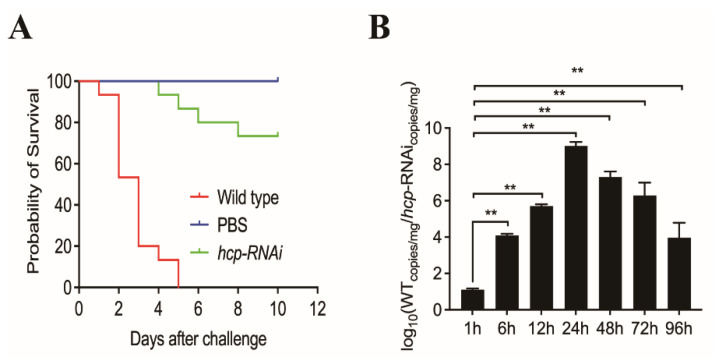
Effects of *hcp* gene silencing on the pathogenicity of *A. salmonicida*. (**A**) To assess the importance of *hcp* to *A. salmonicida* pathogenesis, *E. coioides* were infected with *A. salmonicida* wild-type and *hcp*-RNAi strains. The number of *E. coioides* that survived after infection with the indicated strains was compared (*n* = 3). (**B**) The bacterial burdens of *A. salmonicida* wild-type and *hcp*-RNAi in *E. coioides* spleens were measured by quantitative real-time PCR (qRT-PCR) at 1, 6, 12, 24, 48, 72, and 96 hpi. The temporal dynamic distribution of *A. salmonicida* in *E. coioides* spleens was compared. Data are shown as mean ± SD from three independent biological replicates. ** *p* < 0.01.

To assess the importance of the *hcp* gene for the pathogenesis of *A. salmonicida*, we infected *E. coioides* separately with wild-type and *hcp*-RNAi strains. The results showed that infection with the *hcp*-RNAi strain resulted in significantly delayed death and a lower mortality rate in *E. coioides* compared with the wild-type strain infection group (Figure 5A). The initial death was delayed by 3 days, and the cumulative mortality was reduced by 70%. The colonization of wild-type and *hcp*-RNAi strains in the spleen at 1, 6, 12, 24, 48, 72, and 96 hpi were detected by qRT-PCR. The load of the *hcp*-RNAi strain in the spleen was significantly lower than that of the wild-type strain for most of the post-infection duration (Figure 5B). These results suggested that *hcp* might be involved in the virulence regulation mechanism of *A. salmonicida*, and that it plays an important role in the virulence of *A. salmonicida*.

### 3.4. SDS-PAGE Analysis of the Effect of Hcp on the Protein Composition of Extracellular Products

**Figure 6:**
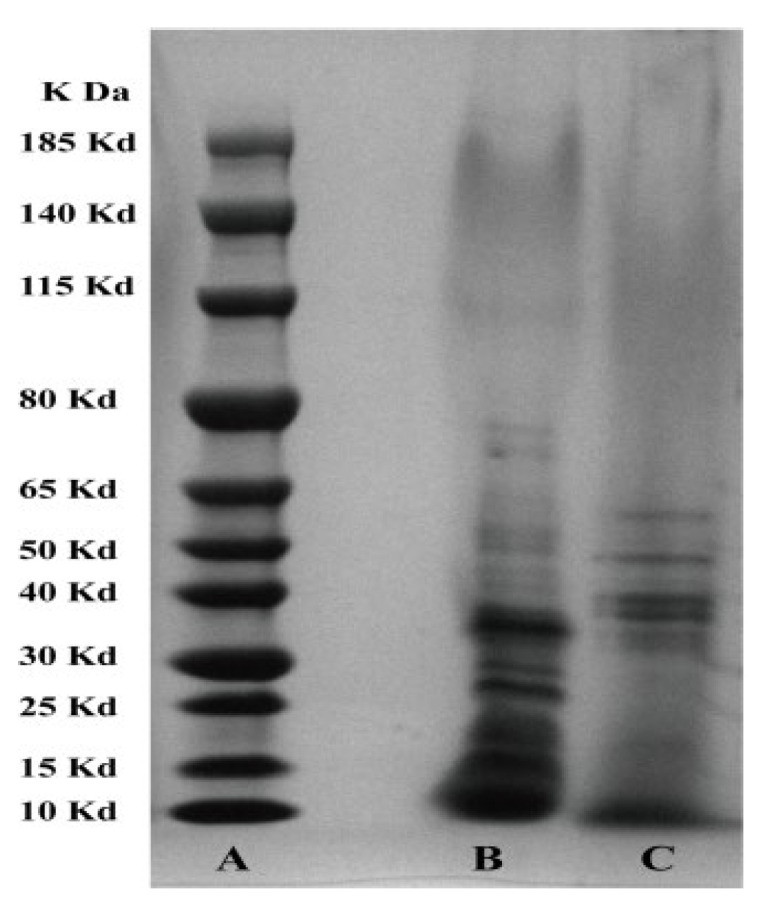
SDS-PAGE protein electropherogram to detect the protein composition of extracellular products of *A. salmonicida*. Note: A—protein marker; B—wild-type strain; C—*hcp*-RNAi strain.

It can be seen from Figure 6 that *hcp* had a great influence on the protein components in the extracellular products of *A. salmonicida*. Compared with the wild-type strain, the protein components in the extracellular product of the *hcp*-RNAi strain were mainly distributed in the range 26–48 kDa, while the bands between 13 and 22 kDa were less represented (Figure 6). It can be seen that after *hcp* gene silencing, the protein components in the extracellular product of *A. salmonicida* changed significantly. This indicated that *hcp* might affect the secretion of virulence factors from *A. salmonicida*, thus having an important influence on the pathogenic mechanism of *A. salmonicida*. Data are presented as mean ± SD. Three independent biological replicates were performed per group. * *p* < 0.05, ** *p* < 0.01.

### 3.5. Effect of Hcp Gene on the Enzyme Activity of A. salmonicida EPS

**Figure 7:**
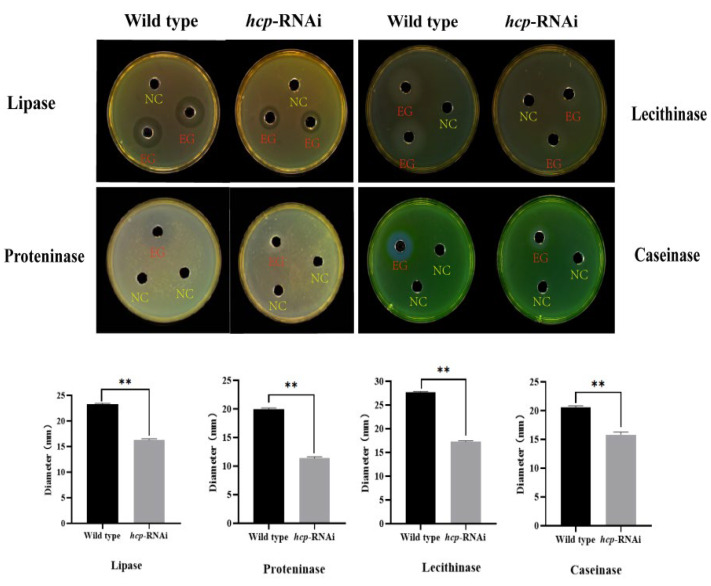
Effects of *hcp* gene silencing on the activity of extracellular enzymes of *A. salmonicida*. Note: NC—negative control; EG—experimental group. Data are presented as mean ± SD. Three independent biological replicates were performed per group. ** *p* < 0.01.

EPSs are also one of the most important pathogenic factors of *A. salmonicida*. We used the Oxford cup method to determine the effect of *hcp* on the enzymatic activity of the extracellular products of *A. salmonicida*. The results showed that there were significant differences in the transparent circles formed around the Oxford cup on the sterile casein (0.4%), nonfat dry milk (0.4%), Tween 80 (1.0%), and sterile egg yolk (2.5%) plates (Figure 7). As shown in Figure 7, the enzymatic activity of the extracellular product of the *hcp*-RNAi strain was significantly reduced compared to that of the wild-type strain. The results showed that *hcp* affected the activities of lipase, lecithinase, proteninase, and caseinase in the extracellular products of *A. salmonicida*.

### 3.6. Effects of Hcp Silencing on EPS Content of A. salmonicida

**Figure 8:**
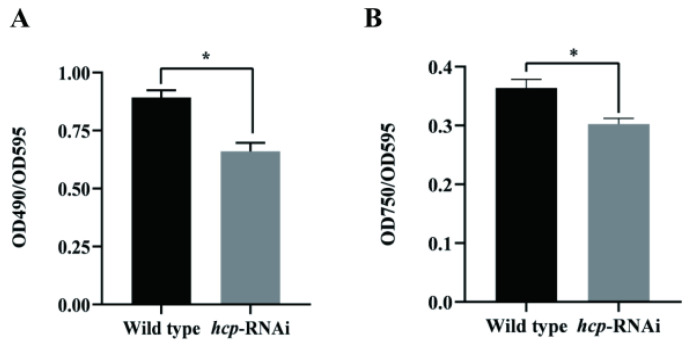
Effects of hcp gene silencing on the content of extracellular polysaccharides (**A**) and extracellular proteins (**B**) in *A. salmonicida*. Data are presented as mean ± SD. Three independent biological replicates were performed per group. * *p* < 0.05.

EPSs mainly include exopolysaccharides and extracellular proteins. In order to explore the effect of *hcp* on the EPSs of *A. salmonicida*, we determined the relative content changes of the exopolysaccharides and extracellular proteins of the wild-type and *hcp*-RNAi strains using the phenol sulfuric acid method and Lowry reagent, respectively. As can be seen from Figure 8, compared with the wild-type strain, the contents of exopolysaccharides and extracellular proteins in the *hcp*-RNAi strain were significantly reduced, indicating that *hcp* affected the content of exopolysaccharides and extracellular proteins in *A. salmonicida*.

## 4. Discussion

In order to compete for living space and nutrients, bacteria have evolved a contact inhibition mechanism [52]: for example, the contact-dependent growth inhibition system and T6SS reported in *E. coli* and *V. cholerae* [53,54]. As an important member of T6SS, Hcp was first discovered and named by Williams et al. in the regulation of virulence gene expression in *V. cholerae* [54]. Previous studies have shown that Hcp is closely related to bacterial virulence and QS regulation and plays an important role in bacterial survival and pathogenesis [25,54,55,56]. The results of the whole-genome sequencing of *A. salmonicida* by Huang et al. showed that *A. salmonicida* had an entire T6SS [57]; however, so far there has been no report on the *A. salmonicida hcp* gene and its related functions. In this study, through sequence alignment and the phylogenetic tree analysis of the amino acid sequence of *A. salmonicida* Hcp, we found that Hcp was highly conserved among *A. salmonicida*, which belongs to the same family as *A. hydrophila*, and had high homology.

T6SS plays an important role in the survival and pathogenesis of Gram-negative bacteria [58]. In fact, Hcp is a highly conserved component of T6SS, and bacteria containing T6SS secrete Hcp under suitable conditions, which has been used as a reliable indicator for confirming T6SS [18]. In order to better understand the structure of Hcp, we compared the results of the I-TASSER protein structure prediction and found that the Hcp of *A. salmonicida* had the highest similarity with the Hcp protein structure model of *V. cholerae* (PDBID: 5mxn1; Figure 2A,B), and the amino acid sequence homology of the Hcp of the two bacteria was 78.49%. The Hcp of *V. cholerae* folds into a β-sheet roll, and each Hcp hexameric loop consists of a total of 24 β-strands on the inner surface (Figure 2B, indicated by the purple arrow) [59]. Multiple Hcp monomers are stacked to form a transport pipeline, which is responsible for transporting effector factors and has the function of a molecular chaperone. It is similar in structure to gp19 of the T4 phage and can be assembled to form a secretion tail tube. At the same time, the Hcp ring growth duct can penetrate the bacterial outer membrane [19,60,61]. When the six Hcp monomers are assembled into a ring-shaped pipeline, they are stacked on top of each other, following the same helical symmetry as the surrounding sheath and forming a hexameric ring to allow the effector substances to pass through and protect them from being degraded [59,60]. The attachment helix of VipB is in contact with the Hcp loop at the edge of the hexamer, and the model salmonicide Hcp has a similar structure (Figure 2E). Proteins with high structural similarity tend to have similar functions in relation to their targets [62]. Therefore, we speculated that after the Hcp of *A. salmonicida* forms a transmembrane hexameric channel, it can be transported to the periplasmic space through the inner membrane protein complex and then interact with the host cell, thereby affecting cell function. At the same time, our studies showed that after the *hcp* gene was silenced, the hemolytic ability and pathogenicity of *A. salmonicida* was significantly reduced (Figure 4B and Figure 5A). In addition, COFACTOR and COACH were used to predict the biological function of *A. salmonicida* Hcp structure and the conservation of active sites, including the ligand-binding sites and enzyme commission number (Figure 2C,D). It was found that the functional active sites of Hcp were highly conserved. In conclusion, we speculate that Hcp plays an important role in the pathogenicity of *A. salmonicida*.

Like most pathogenic bacteria, the virulence factors of *A. salmonicida* are also closely regulated by genes. In order to further explore the pathogenic mechanism of *hcp* in *A. salmonicida*, we constructed stable *hcp*-RNAi strains and studied their biological characteristics. Our results demonstrated that the silencing of the *hcp* gene negatively regulated the adhesion, hemolysis, and biofilm-forming abilities of *A. salmonicida*. The adhesion to and invasion of host cells by bacteria are two key processes in their colonization of the host. T6SS has been shown to be involved in the regulation of biofilm formation and adhesion [63,64]. At the same time, the expression of T6SS is of great significance for the bacterial adhesion to and invasion of host cells, which can affect a pathogen’s ability to infect the host or the severity of a disease. Wang et al. found that *hcp*1, *hcp*2, and *hcp*3 were all involved in the biofilm formation of *A. hydrophila*, and that *hcp*1 and *hcp*3 had a positive effect on biofilm formation, while *hcp*2 had the opposite effect [65]. Yu et al. found that after the deletion of the T6SS2 structural protein gene ivmF2 and the translocation protein gene *hcp*2 in *Vibrio parahaemolyticus*, the adhesion ability of *V. parahaemolyticus* to Hela cells decreased [66]. In addition, Nancy et al. showed that T6SS caused the contact-dependent lysis of erythrocytes in Campylobacter jejuni, and the hemolytic capacity was significantly reduced in *hcp* and *tssM* mutants [67].

During the growth and reproduction of pathogenic bacteria, cells continuously release metabolites into the environment, and these metabolites are extremely important for the absorption of nutrients by cells and immune evasion. In addition, some extracellular products, such as extracellular protease, and hemolysin can directly destroy the host’s constitution, causing the host to become morbid and even die [68]. Our results showed that *hcp* silencing changed the protein components in the extracellular products of *A. salmonicida* compared with the wild-type strain, while the activities of some enzymes, such as protease, lipase, lecithinase, and caseinase, were significantly reduced. At the same time, some studies have shown that the pathogenicity of extracellular products can reflect the virulence of pathogenic bacteria [69].

The pathogenicity of bacteria is not achieved by a single virulence factor, but by the joint action of multiple virulence factors [70,71]. In our study, we found that *hcp* silencing affected important virulence factors of *A. salmonicida* such as biofilm formation, adhesion, and extracellular product enzymatic activity. Our results showed that compared with the wild-type strain, the bacterial colonization in the spleens of *E. coioides* was reduced in the *hcp*-RNAi strain infection group, the initial death of *E. coioides* was delayed by 3 days, and the cumulative mortality was significantly reduced. This was consistent with the trend of the *hcp*-deletion strain reported in porcine extraintestinal pathogenic *E. coli* by Ying et al. [72]. Studies have shown that bacterial T6SS has a variety of biological functions. By mediating the effect on bacteria or eukaryotic cells, it can affect the pathogenic ability of bacteria and improve the adaptability of bacteria to the environment [73]. Our results indicated that *hcp* is a virulence gene of *A. salmonicida* and plays an important role in the infection process.

Summary: As an important structural protein and effector protein, Hcp plays a vital role in T6SS. Based on the above research, we found that the Hcp of *A. salmonicida* (strain: SRW-OG1) has the highest similarity with the Hcp structure model of *V. cholerae*, and the amino acid sequence homology of the Hcp of the two bacteria was determined to be 78.49%. At the same time, we found that *hcp* is involved in the biofilm formation, adhesion, hemolysis, extracellular product enzymatic activity, and extracellular protein and exopolysaccharide secretion of *A. salmonicida*, indicating that *hcp* is an important virulence gene. The results of this study could lay a foundation for further research on the biological function of *A. salmonicida* Hcp, an in-depth study of the mechanisms of T6SS in the pathogenic process of *A. salmonicida*, and the search for effective control methods for *A. salmonicida* infection.

## Supplementary Materials

The following supporting information can be downloaded at: https://www.mdpi.com/article/10.3390/microorganisms10122307/s1, Table S1: Sequences of primers used in this study.

## References

[1] Jin S., Fu S. (2020). Identification and histopathological and pathogenicity analysis of *Aeromonas salmonicida* salmonicida from goldfish (*Carassius auratus*) in North China. Aquac. Fish..

[2] Van Banning P. (1987). Bacterial Fish Pathogens: Disease in Farmed and Wild Fish: B. Austin and DA Austin.

[3] Bricknell I.R., Bowden T.J. (1999). Susceptibility of *Atlantic halibut*, *Hippoglossus hippoglossus* (L.) to infection with typical and atypical *Aeromonas salmonicida*. Aquaculture.

[4] Magariños B., Devesa S. (2011). Furunculosis in *Senegalese sole* (*Solea senegalensis*) cultured in a recirculation system. Vet. Rec.-Engl. Ed..

[5] Lago E.P., Nieto T.P. (2012). Virulence factors of *Aeromonas salmonicida* subsp. salmonicida strains associated with infections in turbot Psetta maxima. Dis. Aquat. Org..

[6] Briones V., Fernandez A. (1998). Haemorrhagic Septicaemia by *Aeromonas salmonicida* subsp. salmonicida in a Black-tip Reef Shark (*Carcharhinus melanopterus*). J. Vet. Med. Ser. B.

[7] Burr S.E., Pugovkin D. (2005). Attenuated virulence of an *Aeromonas salmonicida* subsp. salmonicida type III secretion mutant in a rainbow trout model. Microbiology.

[8] Orozova P., Barker M. (2009). Identification and pathogenicity to rainbow trout, *Oncorhynchus mykiss* (Walbaum), of some aeromonads. J. Fish Dis..

[9] Marcoux P.É., Vincent A.T. (2020). Systematic analysis of the stress-induced genomic instability of Type Three Secretion System in *Aeromonas salmonicida* subsp. salmonicida. Microorganisms.

[10] Balado M., Souto A. (2015). Two catechol siderophores, acinetobactin and amonabactin, are simultaneously produced by *Aeromonas salmonicida* subsp. salmonicida sharing part of the biosynthetic pathway. ACS Chem. Biol..

[11] Schwenteit J., Gram L. (2011). Quorum sensing in *Aeromonas salmonicida* subsp. achromogenes and the effect of the autoinducer synthase AsaI on bacterial virulence. Vet. Microbiol..

[12] Garduño R.A., Moore A.R. (2000). Host cell invasion and intracellular residence by *Aeromonas salmonicida*: Role of the S-layer. Can. J. Microbiol..

[13] Daher R.K., Filion G. (2011). Alteration of virulence factors and rearrangement of pAsa5 plasmid caused by the growth of *Aeromonas salmonicida* in stressful conditions. Vet. Microbiol..

[14] Carniel E., Guilvout I. (1996). Characterization of a large chromosomal “high-pathogenicity island” in biotype 1B *Yersinia enterocolitica*. J. Bacteriol..

[15] Russell A.B., Peterson S.B. (2014). Type VI secretion system effectors: Poisons with a purpose. Nat. Rev. Microbiol..

[16] Cherrak Y., Flaugnatti N. (2019). Structure and activity of the type VI secretion system. Microbiol. Spectr..

[17] Bröms J.E., Meyer L. (2012). DotU and VgrG, core components of type VI secretion systems, are essential for Francisella LVS pathogenicity. PLoS ONE.

[18] Boyer F., Fichant G. (2009). Dissecting the bacterial type VI secretion system by a genome wide in silico analysis: What can be learned from available microbial genomic resources?. BMC Genom..

[19] Silverman J.M., Agnello D.M. (2013). Haemolysin coregulated protein is an exported receptor and chaperone of type VI secretion substrates. Mol. Cell.

[20] Hachani A., Wood T.E. (2016). Type VI secretion and anti-host effectors. Curr. Opin. Microbiol..

[21] Sha J., Rosenzweig J.A. (2013). Evaluation of the roles played by Hcp and VgrG type 6 secretion system effectors in *Aeromonas hydrophila* SSU pathogenesis. Microbiology.

[22] Manera K., Caro F. (2021). Sensing of intracellular Hcp levels controls T6SS expression in *Vibrio cholerae*. Proc. Natl. Acad. Sci. USA.

[23] Zhou Y., Tao J. (2012). Hcp family proteins secreted via the type VI secretion system coordinately regulate *Escherichia coli* K1 interaction with human brain microvascular endothelial cells. Infect. Immun..

[24] Carruthers D., Nicholson A. (2013). *Acinetobacter baumannii* utilizes a type VI secretion system for bacterial competition. PLoS ONE.

[25] Huang L., Qi W. (2020). The immune response of a warm water fish orange-spotted grouper (*Epinephelus coioides*) infected with a typical cold water bacterial pathogen *Aeromonas salmonicida* is AhR dependent. Dev. Comp. Immunol..

[26] Zhong Y., Qi W. (2021). Insights into mesophilic virulence, antibiotic resistant and human pathogenicity: A genomics study on the *Aeromonas salmonicida* SRW-OG1 newly isolated from the Asian fish *Epinephelus coioides*. Aquaculture.

[27] Saitou N., Nei M. (1987). The neighbor-joining method: A new method for reconstructing phylogenetic trees. Mol. Biol. Evol..

[28] Yang J., Roy A. (2012). BioLiP: A semi-manually curated database for biologically relevant ligand–protein interactions. Nucleic Acids Res..

[29] Wu S., Zhang Y. (2007). LOMETS: A local meta-threading-server for protein structure prediction. Nucleic Acids Res.

[30] Zhang Y., Kihara D. (2002). Local energy landscape flattening: Parallel hyperbolic Monte Carlo sampling of protein folding. Proteins.

[31] Li Y., Zhang Y. (2009). REMO: A new protocol to refine full atomic protein models from C-alpha traces by optimizing hydrogen-bonding networks. Proteins.

[32] Zhang J., Liang Y. (2011). Atomic-level protein structure refinement using fragment-guided molecular dynamics conformation sampling. Structure.

[33] Roy A., Xu D. (2011). A protocol for computer-based protein structure and function prediction. J. Vis. Exp..

[34] Webb E.C. (1992). Enzyme Nomenclature 1992: Recommendations of the Nomenclature Committee of the International Union of Biochemistry and Molecular Biology on the Nomenclature and Classification of Enzymes.

[35] Luo G., Huang L. (2016). flrA, flrB and flrC regulate adhesion by controlling the expression of critical virulence genes in *Vibrio alginolyticus*. Emerg. Microbes Infect..

[36] Sun Q., Liu Y., Teng X., Luan P., Teng X., Yin X. (2022). Immunosuppression participated in complement activation-mediated inflammatory injury caused by 4-octylphenol via TLR7/IκBα/NF-κB pathway in common carp (*Cyprinus carpio*) gills. Aquat. Toxicol..

[37] Cui J., Zhou Q., Yu M., Liu Y., Teng X., Gu X. (2022). 4-tert-butylphenol triggers common carp hepatocytes ferroptosis via oxidative stress, iron overload, SLC7A11/GSH/GPX4 axis, and ATF4/HSPA5/GPX4 axis. Ecotoxicol. Environ. Saf..

[38] Choi K.H., Schweizer H.P. (2006). mini-Tn7 insertion in bacteria with single attTn7 sites: Example *Pseudomonas aeruginosa*. Nat. Protoc..

[39] Darsigny M., Babeu J.-P. (2010). Hepatocyte nuclear factor-4α promotes gut neoplasia in mice and protects against the production of reactive oxygen species. Cancer Res..

[40] Zuo Y., Zhao L. (2019). Mechanisms underlying the virulence regulation of new *Vibrio alginolyticus* ncRNA Vvrr1 with a comparative proteomic analysis. Emerg. Microbes Infect..

[41] Hu L., Zhao L. (2021). The Effect of tonB Gene on the Virulence of *Pseudomonas plecoglossicida* and the Immune Response of *Epinephelus coioides*. Front. Microbiol..

[42] Mao L., Qin Y. (2020). Role of LuxR-type regulators in fish pathogenic *Aeromonas hydrophila*. J. Fish Dis..

[43] Huang L., Huang L. (2016). The TCA pathway is an important player in the regulatory network governing *Vibrio alginolyticus* adhesion under adversity. Front. Microbiol..

[44] He R., Zuo Y. (2021). Copper stress by nutritional immunity activates the CusS-CusR two-component system that contributes to *Vibrio alginolyticus* anti-host response but affects virulence-related properties. Aquaculture.

[45] Tsou A.M., Zhu J. (2010). Quorum sensing negatively regulates hemolysin transcriptionally and posttranslationally in *Vibrio cholerae*. Infect. Immun..

[46] Huang L., Zhang Y. (2019). Phenotypic characterization, virulence, and immunogenicity of *Pseudomonas plecoglossicida* rpoE knock-down strain. Fish Shellfish. Immunol..

[47] Huang L., Zuo Y. (2021). The Zinc Nutritional Immunity of Epinephelus Coioides Contributes to the Importance of znuC During *Pseudomonas plecoglossicida* Infection. Front. Immunol..

[48] Lutwyche P., Exner M.M. (1995). A conserved *Aeromonas salmonicida* porin provides protective immunity to rainbow trout. Infect. Immun..

[49] Yuan Y., Li J. (2021). Extracellular products-mediated interspecific interaction between *Pseudomonas aeruginosa* and *Escherichia coli*. J. Microbiol..

[50] Kim H.S., Park H.D. (2013). Ginger extract inhibits biofilm formation by *Pseudomonas aeruginosa* PA14. PLoS ONE.

[51] Huang L., Zuo Y. (2019). A metabolomic investigation into the temperature-dependent virulence of *Pseudomonas plecoglossicida* from large yellow croaker (*Pseudosciaena crocea*). J. Fish Dis..

[52] Hayes S., Aoki K. (2010). Bacterial contact-dependent delivery systems. Annu. Rev. Genet..

[53] Williams G., Varcoe T. (1996). *Vibrio cholerae* Hcp, a secreted protein coregulated with HlyA. Infect. Immun..

[54] Pukatzki S., Ma T. (2006). Identification of a conserved bacterial protein secretion system in *Vibrio cholerae* using the Dictyostelium host model system. Proc. Natl. Acad. Sci. USA.

[55] Zheng J., Leung Y. (2007). Dissection of a type VI secretion system in *Edwardsiella tarda*. Mol. Microbiol..

[56] Mougous J.D., Cuff M.E. (2006). A virulence locus of *Pseudomonas aeruginosa* encodes a protein secretion apparatus. Science.

[57] Roy A., Kucukural A. (2010). I-TASSER: A unified platform for automated protein structure and function prediction. Nat. Protoc..

[58] Russel B., Hood D. (2011). Type VI secretion delivers bacteriolytic effectors to target cells. Nature.

[59] Wang J., Brackmann M. (2017). Cryo-EM structure of the extended type VI secretion system sheath–tube complex. Nat. Microbiol..

[60] Gallique M., Bouteiller M. (2017). The Type VI Secretion System: A Dynamic System for Bacterial Communication?. Front. Microbiol..

[61] Pukatzki S., McAuley S.B. (2009). The type VI secretion system: Translocation of effectors and effector-domains. Curr. Opin. Microbiol..

[62] Kuhlman B., Bradley P. (2019). Advances in protein structure prediction and design. Nat. Rev. Mol. Cell Biol..

[63] Aubert F., Flannagan S. (2008). A novel sensor kinase-response regulator hybrid controls biofilm formation and type VI secretion system activity in *Burkholderia cenocepacia*. Infect. Immun..

[64] Liu L., Hao S. (2015). The Type VI Secretion System Modulates Flagellar Gene Expression and Secretion in *Citrobacter freundii* and Contributes to Adhesion and Cytotoxicity to Host Cells. Infect. Immun..

[65] Wang N., Liu J. (2018). Diverse roles of Hcp family proteins in the environmental fitness and pathogenicity of *Aeromonas hydrophila* Chinese epidemic strain NJ-35. Appl. Microbiol. Biotechnol..

[66] Yu Y., Fang L. (2015). VgrG2 of type VI secretion system 2 of *Vibrio parahaemolyticus* induces autophagy in macrophages. Front. Microbiol..

[67] Bleumink-Pluym N.M., van Alphen L.B. (2013). Identification of a functional type VI secretion system in *Campylobacter jejuni* conferring capsule polysaccharide sensitive cytotoxicity. PLoS Pathog..

[68] Labella A.M., Rosado J.J. (2020). Virulence properties of three new Photobacterium species affecting cultured fish. J. Appl. Microbiol..

[69] Santos Y., Bandin I. (1992). Comparison of the extracellular biological activities of *Vibrio anguillarum* and *Aeromonas hydrophila*. Aquaculture.

[70] Li J., Ni X. (2011). Detection of three virulence genes alt, ahp and aerA in *Aeromonas hydrophila* and their relationship with actual virulence to zebrafish. J. Appl. Microbiol..

[71] Zhou Q.L., Wang Y. (2013). Distribution and virulence gene comparison of *Aeromonas* strains isolated from diseased fish and water environment. Pol. J. Microbiol..

[72] Peng Y., Wang X. (2016). Roles of Hcp family proteins in the pathogenesis of the porcine extraintestinal pathogenic *Escherichia coli* type VI secretion system. Sci. Rep..

[73] Zhao W., Caro F. (2018). Antagonism toward the intestinal microbiota and its effect on *Vibrio cholerae* virulence. Science.

