# Supplementary material for: Effect of the Type VI Secretion System Secreted Protein Hcp on the Virulence of Aeromonas salmonicida
Source: Microorganisms. 2022 Nov 22;10(12):2307. doi: 10.3390/microorganisms10122307 (PMC9784854; doi:10.3390/microorganisms10122307)
Supplement: Supplementary file 1 [file microorganisms-10-02307-s001.zip › Supplementary Material Table S1.pdf]

Supplementary Material Table S1. qRT-PCR primer sequences

Primer Name	Primers sequence for the qRT-PCR（5’→3’）
hcp	F：CAAGCAGGAGCACTTTTTC
	R：CTGTGTGTTCCCAGTCAAT
16*S*	F：TTGATTTACACTCCTCGCCTAAC
	R：CAGCCAACTTGCCGTACTCT
